# Optimized models for design of efficient miR30-based shRNAs

**DOI:** 10.3389/fgene.2012.00163

**Published:** 2012-08-29

**Authors:** Olga V. Matveeva, Nafisa N. Nazipova, Aleksey Y. Ogurtsov, Svetlana A. Shabalina

**Affiliations:** ^1^Department of Human Genetics, University of UtahSalt Lake City, UT, USA; ^2^Novosibirsk State UniversityNovosibirsk, Russia; ^3^Institute of Mathematical Problems of Biology, Russian Academy of SciencesPushchino, Russia; ^4^National Center for Biotechnology Information, National Library of Medicine, National Institutes of HealthBethesda, MD, USA

**Keywords:** shRNA design, computational models, thermodynamic parameters, miR30-based shRNA

## Abstract

Small hairpin RNAs (shRNAs) became an important research tool in cell biology. Reliable design of these molecules is essential for the needs of large functional genomics projects. To optimize the design of efficient shRNAs, we performed comparative, thermodynamic, and correlation analyses of ~18,000 miR30-based shRNAs with known functional efficiencies, derived from the Sensor Assay project (Fellmann et al., 2011). We identified features of the shRNA guide strand that significantly correlate with the silencing efficiency and performed multiple regression analysis, using 4/5 of the data for training purposes and 1/5 for cross validation. A model that included the position-dependent nucleotide preferences was predictive in the cross-validation data subset (*R* = 0.39). However, a model, which in addition to the nucleotide preferences included thermodynamic shRNA features such as a thermodynamic duplex stability and position-dependent thermodynamic profile (dinucleotide free energy) was performing better (*R* = 0.53). Software “miR_Scan” was developed based upon the optimized models. Calculated mRNA target secondary structure stability showed correlation with shRNA silencing efficiency but failed to improve the model. Correlation analysis demonstrates that our algorithm for identification of efficient miR30-based shRNA molecules performs better than approaches that were developed for design of chemically synthesized siRNAs (*R*_max_ = 0.36).

## Introduction

RNA interference (RNAi) is a biologically important mechanism of regulation of gene expression by endogenous microRNAs (miRNAs) that silence genes through complementary interaction with their mRNA targets. Precursors of miRNAs (pre-miRNAs) are stable hairpins which are encoded in plant and animal genomes and processed into short duplexes by enzymatic machinery of the cell (see reviews Du and Zamore, 2007; Shabalina and Koonin, 2008). The relative duplex instability at the 5′ end of the RNA guide strand facilitates its preferential incorporation into the RNA Inducible Silencing Complex (RISC) (Khvorova et al., 2003; Schwarz et al., 2003). This mechanism of gene silencing is used in a promising method of suppressing gene expression in eukaryotic cells by endogenously supplied artificial molecules: siRNAs (chemically synthesized short interfering RNA duplexes that can be transfected into cells) and shRNAs (small hairpin RNAs that can be expressed in cells from endogenously supplied plasmids or viral vectors) (Brummelkamp et al., 2002). Recent studies suggest that siRNAs and shRNAs are functionally interchangeable with miRNAs, and the choice of mRNA translational repression versus mRNA cleavage is determined solely by the degree of complementarity between small RNAs and their targets (Hutvagner and Zamore, 2002; Doench et al., 2003; Tang, 2005). Similar to miRNAs, exogenous shRNAs are processed in the cell by nucleases into short duplexes consisting of the antisense (cleavage guidance) strand and the sense (passenger) strand, where antisense strands are complementary to their RNA targets and specifically silence gene expression.

Similar to miRNAs, shRNAs, and siRNAs are incorporated in and processed by RISCs. The assembly of the RISC is a key step in RNAi. Small RNA duplexes are functionally asymmetric and enhanced flexibility of miRNA precursors at the 5′-antisense terminal base pair was demonstrated in miRNAs and siRNAs (Khvorova et al., 2003). Thermodynamic stability of the base pairs at the 5′ ends of the two siRNA strands differs, which determines the degree to which each strand participates in the RNAi pathway and results in the preferential incorporation of the guide strand into RISC (Schwarz et al., 2003; Tomari et al., 2004). The selective assembly of the antisense strand into RISC probably reflects the relative ease of unwinding from one end of the antisense-sense duplex. The thermodynamic properties of the miRNA-like and siRNA-like duplexes, such as terminal end stability, measured as Gibbs free energy, determine the asymmetrical RISC assembly and, therefore, the efficiency of target gene silencing. Since processing of artificial siRNAs and shRNAs in cells utilizes the main components of cellular RNAi machinery, design of these molecules should allow provision for successful interaction with RISC and mRNA targets.

The shRNA-based approach to gene silencing is more laborious and time consuming, as compared to synthetic siRNAs, but it is becoming increasingly popular. The shRNA approach offers advantages in silencing longevity over synthesized siRNAs and lower costs for genome-wide studies. Lower intra-cellular concentrations achieved through the natural process of transcription of shRNA for extended periods of time can yield more specific silencing effects, compared to synthetic siRNA oligonucleotides transfected into cells.

The accumulation of published experimental gene silencing data promoted development of the various computer models for predicting siRNA efficiency (Saetrom and Snove, 2004; Huesken et al., 2005; Yiu et al., 2005; Gong et al., 2006; Jia et al., 2006; Shabalina et al., 2006; Takasaki et al., 2006; Birmingham et al., 2007; Jiang et al., 2007; Ladunga, 2007; Matveeva et al., 2007, 2010). The processing of shRNAs with long stems (22 nucleotides or more) depends on Dicer activity, and some popular designs of miRNA-like shRNAs are based on Dicer- and Drosha-mediated RNA cleavage and are used for the loss-of-function assays (Schlabach et al., 2008; Silva et al., 2008). Current predictive models of siRNA behavior are frequently used for shRNA design. However, many of them fail to discriminate between efficient and inefficient shRNAs (Taxman et al., 2006). Earlier we used a combination of different compositional and thermodynamic characteristics for prediction of efficient siRNAs in a number of computational models (Shabalina et al., 2006; Matveeva et al., 2007, 2010). We found that both thermodynamic and compositional features contributed to efficient siRNA design. In this work, we performed comprehensive evaluation of the predictive power of different sequence and thermodynamic parameters and applied our published feature selection procedure (Shabalina et al., 2006) to selection of efficient shRNA molecules. In this study we optimized parameters for predicting the silencing efficiency of shRNAs and identified universal features for both siRNAs and shRNAs. Also it is shown that incorporation of shRNA-specific parameters in the models improved prediction results meaningfully for this type of molecules.

## Materials and methods

### Database

The shRNA set was compiled from published experiments targeting nine distinct mRNAs using a massively parallel sensor assay (Fellmann et al., 2011). In this study thousands of synthetic RNAi triggers were evaluated in functional assays by placing a cognate target site (sensor) in the 3′UTR of a reporter gene and quantifying its RNAi-mediated repression. We obtained shRNA sequences and silencing activities from the Supplementary Materials of the publication by Fellmann et al. (2011) and compiled a database for 18,719 RNAi reporters covering every possible target site in nine complete mammalian transcripts. Values of reported activity are ranged from 0 (no effect) to 100% (complete knock-out).

We used 4/5 of the 18719 shRNAs as a training set. The training set was used for parameter selection and model optimization, such as creation of summarized position-dependent consensus of nucleotide preferences/avoidance. The remaining shRNA sequences were used as a validation set for efficiency predictions employing linear regression weights obtained with the training set. We also used validation set predictions to generate ROC curves for the corresponding classification models.

### Data analysis and feature selection

We calculated a number of thermodynamic features, such as ΔG stability values for the sense–antisense shRNA duplexes, shRNA guide strand intra-molecular structure stability, shRNA guide strand inter-molecular dimer stability, local target mRNA stabilities, and stabilities of each two neighboring base pairs in the sense–antisense shRNAs duplexes that represent thermodynamic profiles for each position of shRNA guide strand. These characteristics were estimated based on the RNA–RNA hybridization parameters using a nearest neighbor model described earlier (Xia et al., 1998; Mathews et al., 1999a).

Thermodynamic features, such as ΔG values that are relevant to stabilities of the sense–antisense shRNA duplexes, shRNA guide strand intra-molecular structure stability, shRNA guide strand inter-molecular dimer stability, local target mRNA stabilities, and stabilities of each two neighboring base pairs in the sense-antisense shRNA duplexes were evaluated using the OligoTherm program (Shabalina et al., 2006). OligoHybrid is a tool for calculation of potential targets of complementary interactions between two RNA molecules (Shabalina et al., 2006). Calculations of potential mRNA secondary structures, estimation of the free energy of the local secondary structure, and prediction of oligonucleotide affinity to nucleic acid targets were performed with the Afold program (Ogurtsov et al., 2006). These programs employ the same nearest neighbor thermodynamic parameters as the Mfold program (Mathews et al., 1999b). The programs are available on request. We used complete transcript and the Venus construct sequences (Dr. Christof Fellmann, personal communication) as described in the experiments by Fellmann and co-authors (2011) for mRNA folding prediction and mRNA target opening.

Silencing experiments were categorized according to the duplex stability measured as ΔG. The average amount of remaining relevant protein evaluated as average fluorescence inhibition in the experimental study was calculated for each category. The average silencing score and the number of representatives in each data category are indicated for shRNA datasets. All calculations were performed within feature's intervals with right margins being included.

Nucleotide position-dependent matrices for guide strands of miRNA sequences were recorded with a numerical code (A → 1,0,0,0; T → 0,1,0,0; C → 0,0,1,0; G → 0,0,0,1).

We evaluated predictive power of a number of parameters that were described in the literature and used earlier for siRNA prediction (Huesken et al., 2005; Shabalina et al., 2006). In all, we compiled a list of about 150 parameters, which was too large for effective analysis. We used two criteria for selection of prediction parameters: significant correlation with activity and stability of the correlation. Both criteria were evaluated on the training set only. We required that efficient parameters had a correlation of at least 0.014 with shRNA efficiency, and that this correlation be significant at the 0.05 level. This left us with ~40 parameters, as detailed in Results. Since our training set was heterogeneous, combining experiments for nine different mRNAs, we had the opportunity to select those parameters which are most universal. To do this, we split our data set into *n* parts (*n* = 5 and 10), and computed the correlation coefficient for every part and parameter. Taking 1000 such splits, we computed *S*_*n*_ stability value, the standard deviation of *R* for every parameter. We used *S*_*n*_ stability value as an indicator of how much the parameter's predictive power depends on the choice of the particular subset of the data.

### Regression fitting

In our analysis, we used regression, rather than classification models, since they provide more information, are more flexible, and easier to evaluate. We performed multiple linear regression analysis on our sets of ~40 parameters, with cross-validation as described below. “Analyse it” standard edition software produced by Analyse-it Software, Ltd. (UK) was used for regression fitting. Analysis of mini regression models was performed using Excel software. For all model fitting procedures, logarithm of silencing score was used as a dependant variable. For ROC analysis we used on-line tool http://www.rad.jhmi.edu/jeng/javarad/roc/JROCFITi.html developed by John Eng (Russell H. Morgan Department of Radiology and Radiological Science at Johns Hopkins University).

When choosing or optimizing models on the training set, we used *n*-fold cross-validation, a standard method for evaluating model generalization. Cross-validation randomly splits the data set into *n* equally sized subsets. Then, we trained, in turn, on each subset set of *n*–1, validating on the remaining one. The validation predictions from the *n* models combined to make a prediction for every data point. Using these predictions, we computed the coefficient of determination *R*^2^ = (*Actual Variation* − *Error*)/(*Actual Variation*), where the actual variation is ∑_*i*_(*Actual efficiency*_*i*_ − *Average efficiency*)^2^, and the error is ∑_*i*_(*Actual efficiency*_*i*_ − *Predicted efficiency*_*i*_)^2^. Thus, *R*^2^ reflects the percentage of variation in efficiency explained by our model. If the predictions came from a non-cross-validated linear regression, this *R*^2^ would exactly match the square of Pearson's correlation coefficient. Hence, we did all cross-validation using the simple algorithm above with *n* = 5. We also compared the results of our non-overlapping cross-validation algorithm with standard random cross-validation.

## Results and discussion

### Position-dependent consensus

**Figure 1:**
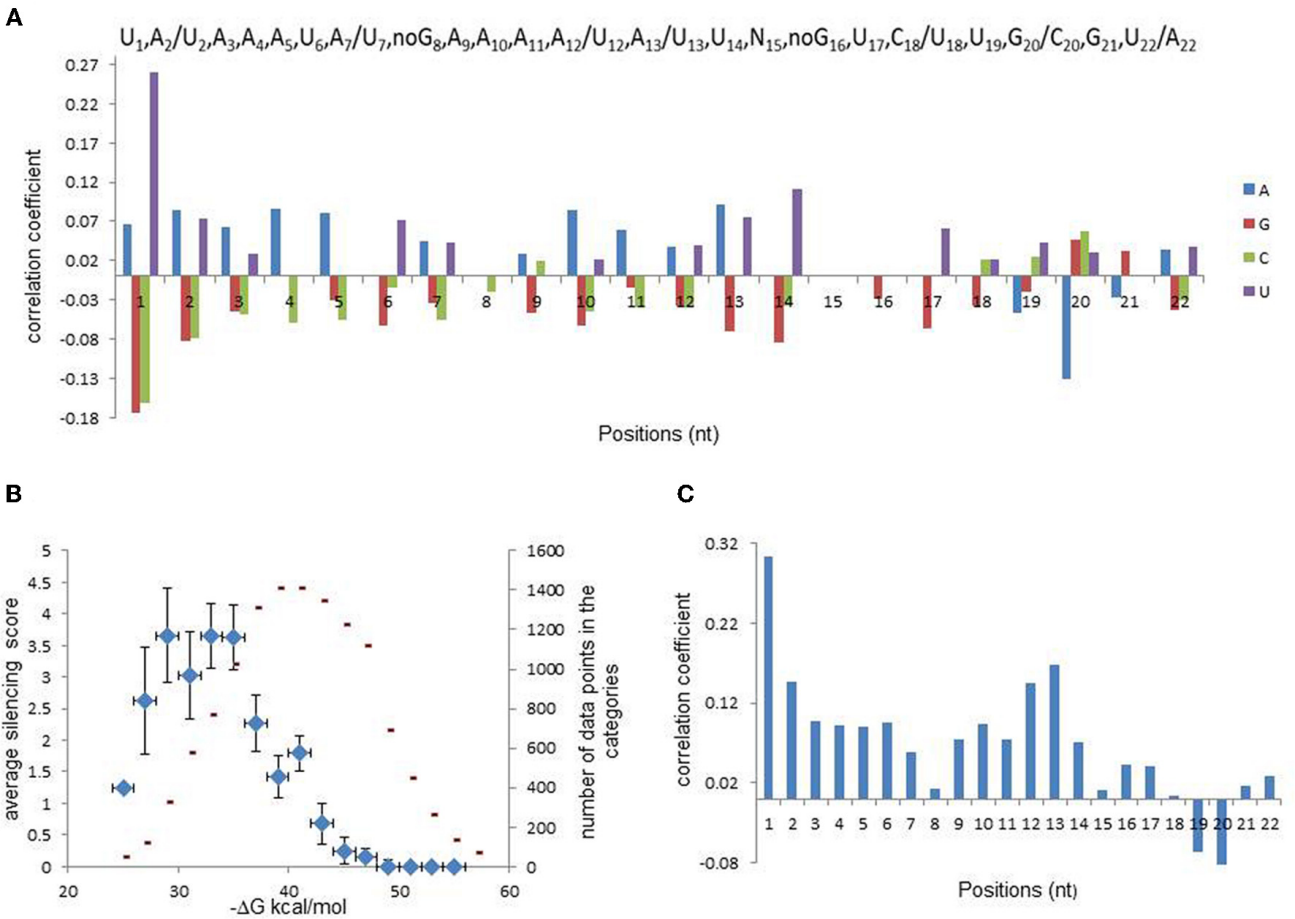
**Analysis of relationships between silencing efficiency and features of shRNA guide strand. (A)** Correlations between shRNA silencing efficiency score (ln) and nucleotide presence at each sequence position (bottom panel). Position-dependent consensus for efficient miR30-based shRNAs (top panel). Two nucleotides were included in the consensus at the same position if correlation coefficients between silencing efficiency and their presence were similar. **(B)** Data categorization according to shRNA duplex stability that shows relationship between this stability and shRNA silencing efficiency. For creation of the scatter-plots, data-points were separated into bines according to total or regional duplex stabilities and average silencing was evaluated for each bin. shRNA silencing efficiency values are shown as blue diamonds and numbers of data-point in each bin are shown in red. Regional or total duplex stability was estimated as total Gibbs free energy, (ΔG), and shRNA silencing efficiency was determined from fluorescence. **(C)** Correlation between silencing efficiency and shRNA local dinucleotide duplex stabilities.

The relationships between the silencing efficiency and nucleotide preferences/avoidances at defined positions of siRNAs were described earlier (Shabalina et al., 2006). In order to find out whether similar relationships exist for miR30-based shRNAs, we recorded oligonucleotide sequences in a numerical code (A → 1,0,0,0; C → 0,1,0,0; G → 0,0,1,0; T → 0,0,0,1). Correlation analysis between the silencing efficiency score and position-dependent matrix for each particular nucleotide at all sequence positions was performed. Figure 1A shows correlation coefficient values that were found to be significant (*P* < 0.05), and complete data are presented in Table A1. Notably, the strongest correlation was detected for U_1_, indicating that the majority of efficient shRNA molecules carry U at the 5′ end of the cleavage guidance strand. It was recently shown that U_1_ and A_1_ are preferentially bound by AGO2 (Frank et al., 2010). Most likely, the 5′-terminal U is important for efficient guide strand entry in RISC (Shabalina et al., 2006; Birmingham et al., 2007). Correlation analysis showed that significant position-dependent bias was also observed for nucleotides located at all positions in the guide strand with the exception of position 15. Most pronounced position-dependent bias was observed for nucleotides located at positions 1 through 7, 10 through 14, 17, 20, and 22.

Considering that the guide strand of efficient molecules carry preferred and avoided nucleotides at specific positions, we compiled position-dependent consensus sequence for the guide strand of efficient shRNAs, which is presented in Figure 1A. This consensus shows preferred nucleotides with the highest values of correlation coefficients between their occurrence and the silencing score. However, when more than one nucleotide correlate with the silencing efficiency significantly (*P* < 0.05), only one of them with the lowest *P*-value is included in the consensus. Thus, the consensus is not providing complete information about all nucleotide preferences related to silencing efficiency, whereas the position-dependent matrix contains the complete information and could be used for optimization of prediction models.

### Silencing efficiency and stability of shRNA fully paired duplex

As shown previously, stability of fully paired duplex formed between siRNA guide strand and target mRNA (estimated as total Gibbs free energy, ΔG duplex) correlates with the silencing efficiency (Shabalina et al., 2006; Ichihara et al., 2007; Matveeva et al., 2007, 2010). To find out whether such a relationship exists for miR30-based shRNAs, data were binned according to ΔG duplex and average silencing score was evaluated for each category. The results of the data processing are presented as a scatter plot in Figure 1B. The scatter plot shows that this thermodynamic characteristic is well described by bell-shaped function, demonstrating non-linear relationship between the silencing efficiency and ΔG duplex for the set of miR30-based shRNAs. The average of the silencing score is growing along with the decrease of ΔG values between −20 and −30 kcal/mol, reaches the maximum value at −33 kcal/mol and declines between −35 and −55 kcal/mol. Figure 1B also shows the frequency distribution of shRNA total ΔG values, with the maximum ΔG values around −40 kcal/mol. Notable, these two distributions do not overlap; the maximum shRNA efficiency (−33 kcal/mol) and the maximum frequency (−40 kcal/mol) occur at different ΔG values. In other words, only minority of all possible scanning constructs possess duplex stability, which is optimal for silencing.

### Silencing efficiency and thermodynamic profile (free energy of paired dinucleotides)

As shown previously, siRNA thermodynamic profiles, evaluated as Gibbs free energy of nearest neighbors (ΔG of dinucleotides) for each paired dinucleotide in a duplex between the guide strand and its fully complemented strand, correlates with the silencing efficiency (Khvorova et al., 2003; Schwarz et al., 2003; Shabalina et al., 2006). Specifically, strong correlations were found for the terminal dinucleotides of the duplex. Stability of some internal dinucleotides in a duplex is also pronounced characteristic for the prediction of the silence efficiency (Shabalina et al., 2006; Matveeva et al., 2007, 2010). To evaluate these correlations in the database of miR30-based shRNA constructs, guide strands of miRNA sequences were recorded in a matrix, where every two neighboring nucleotides were substituted with the nearest neighbor ΔG value. The results of correlation analysis between the silencing efficiency score and local ΔG values are presented in Figure 1C. We found significant (*P* < 0.05) positive and negative correlations for all dinucleotide positions, excluding positions 8, 15, and 18 from the 5′ end of the guide strand (ΔG_8_, ΔG_15_, and ΔG_18_). The strongest and most significant correlation was found for Gibbs free energy of the first dinucleotide (ΔG_1_). Unstable pairing of this dinucleotide with its targets is characteristic of the most efficient silencing molecules and is a prerequisite for efficient guide strand entry into RISC. However, there are several others positions in thermodynamic profiles of the duplex which affect silencing activity. Strong correlation between silencing efficiency and dinucleotide stability was also found for Gibbs free energy of the thirteenth dinucleotide (ΔG_13_). Unstable pairing of this dinucleotide in a duplex is also a characteristic of the most efficient silencing molecules. On the contrary, stable pairing of dinucleotides 19 and 20 with their partners in a duplex is required for silencing efficiency (Figure 1C). Thermodynamic characteristics of miR30-based shRNAs are well described by bell-shaped functions, demonstrating non-linear relationships between the silencing efficiency and ΔG parameters in the seed regions at the 5′ ends of the duplex (positions 2–7) as well as at the 3′ end between positions 15 and 22.

Average silencing efficiency values were calculated according to four separate categories of ΔG (free energy of paired dinucleotide) in each position of duplex thermodynamic profile (see Table A2). Analysis of these values showed that most dinucleotide positions have optimal ΔG values that are connected to highest averaged silencing efficiency within the range of studied ΔG (see Table A2). These data are in a good agreement with previously published thermodynamic profiles of efficient and non-efficient siRNAs (Khvorova et al., 2003; Shabalina et al., 2006) and the notion that optimal stability of the 5′ end of the duplex is significantly lower than that of the 3′ end (*P* < 0.0001).

### Silencing efficiency and thermodynamic features of competitive duplex structures

**Figure A1:**
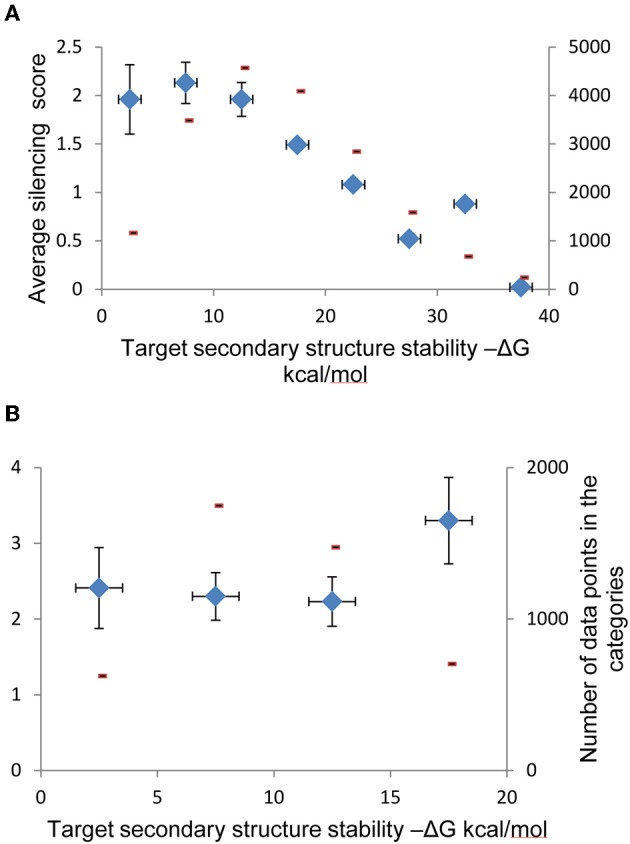
**Categorization of shRNAs according to secondary structure stability of their targets and guide strands. (A)** Data categorization according to target RNA secondary structure stability that shows relationship between this stability and shRNA silencing efficiency in complete dataset. **(B)** Data categorization according to target RNA secondary structure stability that shows relationship between this stability and silencing efficiency among shRNAs with duplex stability (ΔG) between −25 and −35 kcal/mol. Data points were separated into bines according to secondary structure stabilities and average silencing efficiency was evaluated for each bin. Average values for shRNA silencing efficiency are shown in each scatter plot (blue diamonds), along with the numbers of data points in each bin (red marks).

It was shown previously that duplex stability is very important for hybridization and antisense oligo-RNA interaction (Matveeva et al., 2003a,b). It is expected that shRNA intra- or inter-molecular structures can compete with shRNA-target duplex formation which may influence the hybridization efficiency. Some evidence was obtained earlier that the guide siRNA strand secondary structure stability can affect silencing efficiency (Patzel et al., 2005; Köberle et al., 2006; Shabalina et al., 2006). Extensive secondary structure of the target mRNAs can also limit their ability to interact with siRNAs and shRNAs (Overhoff et al., 2005; Shabalina et al., 2006; Shao et al., 2007; Tafer et al., 2008; Matveeva et al., 2010). We addressed the thermodynamics of the mRNA target formation and intra-molecular self-structures of the antisense shRNA strand and found that thermodynamic features significantly correlate (*P* < 0.0001) with the silencing efficiency (Table 1). However, in the subset of the shRNA molecules with optimized duplex stability (ΔG between −35 and −25 kcal/mol) only stability of intra-molecular self-structures of the shRNA guide strand maintained significant correlation with the silencing efficiency (*P* < 0.0001, Table 1), while the stability of the target mRNA secondary structure did not influence significantly the average silencing activity within the ΔG range of duplex between −35 and −25 kcal/mol (Figure A1). Thus, the relative stability of mRNA target formations can be optimized by free energy of the shRNA fully paired duplex (Figure A1). These stability characteristics only slightly improve selection of efficient shRNA targets in mRNAs when they are added to the computational models (data not shown) where influence of the stability of intra-molecular self-structures of the shRNA guide strand is significantly stronger (*P* < 0.0001) than mRNA target stability (*P* < 0.05). The likely explanation to this observation might lie in the experimental conditions where concentration of the shRNA guide strand in the cell is higher than concentration of the target mRNA (Fellmann et al., 2011) due to the difference in transcription efficacy.

**Table 1:** **Correlation analysis between silencing efficiency and free energy of interactions in mRNA targets (target secondary structure stability) or shRNA guide strands (intra- or inter-molecular interactions)**.

	**Pearson correlation**	**Secondary structure and interactions**		
		**Target**	**Guide strand (intra-)**	**Guide strand (inter-)**
Complete database	*R*	0.16	0.13	0.12
	Significance	<10^−100^	1.1 × 10^−72^	2.89 × 10^−56^
Dataset with duplex stability −35 ≤ ΔG ≤ −25 kcal/mol	*R*	–	0.045	–
	Significance	n.s.	0.0018	n.s.

n.s., non-significant correlation (P ≥ 0.05).

### Computational models

In order to identify molecular features that possess predictive power for efficient siRNA design, we analyzed the training set of ~15,000 shRNAs (approximately 4/5 of the total experimental dataset) with experimentally measured activities. To develop silencing prediction model, we performed a linear regression fitting with miR30 sequence features as independent variables and logarithmic values of silencing efficiency scores. We evaluated position dependent nucleotide preferences and avoidances, thermodynamic profiles of shRNA duplexes and the free energy of the fully paired duplex between the sense and antisense strands with these computational models. To analyze nucleotide preferences or avoidances in each position of shRNA antisense strand and to reduce redundancy of the nucleotide position-dependent matrix, we used three (out of four) nucleotides at each position for linear regression (Shabalina et al., 2006). This analysis allowed us to remove from consideration nucleotides with the lowest input to the shRNA efficiency score. Evaluation of preferred and avoided nucleotides at all sequence positions of shRNA training set by multiple regression models included 66 nucleotide variables (22 positions × 3 nucleotides). This multiple regression model showed stable predictive power of shRNA efficiencies (*R* = 0.39). At the next step, we added regression analysis of thermodynamic parameters, such as thermodynamic profiles of shRNA duplexes and free energy of the fully paired duplex between the sense and antisense strands. Taking into account non-linear relationship between the silencing efficiency and free energy of some thermodynamic characteristics, such as free energy of duplex stability and dinucleotide thermodynamic profiles, we used non-linear functions as input in linear regression models (Figure 1B, Tables 2 and A3). When the free energy of shRNA intra-molecular structure formation was added to the computational models it improves the selection of efficient shRNA targets in mRNAs marginally. As discussed earlier inter-molecular structure formation and secondary structure of the target mRNA did not improve the models significantly when added to the computational models (data not shown). This might be explained by the strong correlations between these three parameters and free energy of shRNA fully paired duplex (Mathews et al., 1999a; Shabalina et al., 2006; Matveeva et al., 2010).

**Table 2:** **Statistics and input parameters for the multiple regression model with efficient prediction of silencing activities on the complete database (*n* = 18719 oligonucleotides)**.

**Parameter**	**Coefficient**	***P*-value**
Intercept	−43.85	2.046 × 10^−178^
ΔG	−2.694	4.459 × 10^−111^
ΔG^2^	−0.06917	1.716 × 10^−105^
ΔG^3^	−0.0005959	3.017 × 10^−106^
ΔG_1_	0.3705	0.00048315
A_1_	0.8201	2.61 × 10^−06^
A_2_	0.8225	1.4939 × 10^−11^
A_3_	0.6747	0
A_4_	0.4644	0
A_5_	0.5027	0
A_11_	0.3433	1.0172 × 10^−10^
A_12_	0.4986	0
A_13_	0.9504	0
A_19_	−0.4455	2.1798 × 10^−17^
A_20_	−1.067	2.6228 × 10^−98^
A_22_	0.3039	2.0603 × 10^−08^
G_6_	−0.3063	1.8561 × 10^−08^
G_7_	−0.3133	2.2034 × 10^−08^
G_9_	−0.2439	2.8698 × 10^−06^
G_10_	−0.6907	7.4738 × 10^−28^
G_14_	−0.4776	2.4605 × 10^−18^
G_17_	−0.2477	4.2719 × 10^−06^
G_18_	−0.2378	3.9472 × 10^−06^
G_19_	−0.132	0.0140694
G_21_	0.3678	1.1793 × 10^−12^
C_4_	−0.239	9.663 × 10^−06^
C_5_	−0.3201	3.7375 × 10^−09^
C_7_	−0.4681	3.9052 × 10^−17^
C_8_	−0.1913	0.00023711
C_10_	−0.6128	2.4693 × 10^−22^
C_11_	−0.2256	3.1081 × 10^−05^
C_13_	−0.2326	0.00018904
U_1_	1.989	0
U_2_	0.6581	4.3741 × 10^−07^
U_3_	0.5757	0
U_6_	0.5343	0
U_10_	−0.3281	3.3946 × 10^−08^
U_12_	0.5828	0
U_13_	0.7992	0
U_14_	0.6866	0
U_17_	0.3141	1.7814 × 10^−09^
U_22_	0.3012	2.7612 × 10^−08^

Model fitting was performed using commercially available software “Analyze-it” for Excel.

The complete regression model with 66 nucleotide and 4 thermodynamic variables showed thermodynamic input variables delivered significant weights (Table 2, *P* < 0.001), while some nucleotide input variables were insignificant. We performed a gradual reduction of input variables by removing those that delivered insignificant weights. Each round of reduction involved model refitting. No more than one nucleotide variable per sequence position was removed at each round. This procedure led us to end up with the model with four significant thermodynamic and forty significant nucleotide position-dependent variables and correlation coefficient with experimental silencing score of 0.46. We also calculated stability *S* for these parameters as described previously (Shabalina et al., 2006). We showed that this tight cluster of parameters with *S* ≈ 0.065 as the cut-off (see “Materials and Methods”) is very similar to the stable parameters described for the siRNA design procedure.

**Figure 2:**
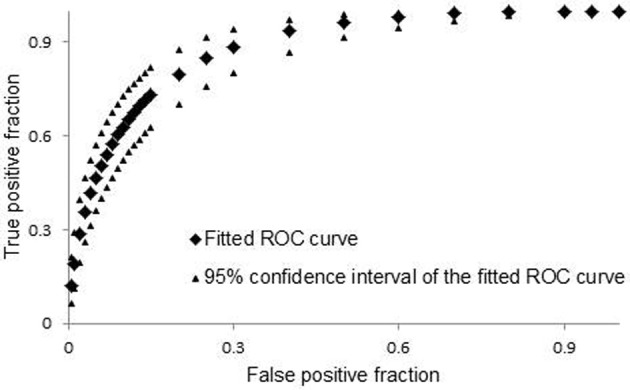
**Cross validation ROC curve.** For ROC analysis we used on-line tool http://www.rad.jhmi.edu/jeng/javarad/roc/JROCFITi.html developed by John Eng (Russell H. Morgan, Department of Radiology and Radiological Science at Johns Hopkins University).

As a part of model cross-validation tests, we performed ROC analysis using binary definition of efficiency for experimental data and continuous model predicted scores for theoretical values. The results of ROC analysis are shown in Figure 2. Total area under the curve (0.882) indicates that our model is predictive and close in predictive ability to the models that were developed for design of siRNA molecules (Matveeva et al., 2007).

**Figure A2:**
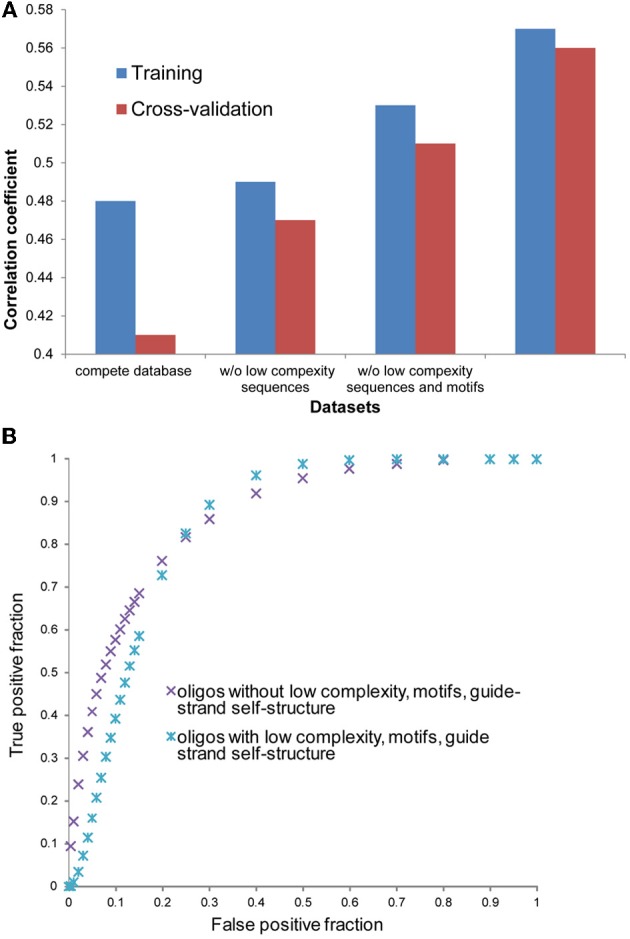
**Correlation analysis with different subsets of oligonucleotides where three different procedures of filtration were applied. (A)** Correlation coefficients for the regression models with the same input parameters, but different variants of datasets for data training. The first dataset variant includes complete database, the second and third datasets includes data without low complexity sequences and without low complexity sequences and motifs, as described in the “Results,” correspondingly. The fourth dataset includes data from the third dataset from which all data points that corresponded to miRNA with guided strand able to form stable secondary structure (ΔG < −2 kcal/mol) were removed. **(B)** Cross validation ROC curves for fourth and control datasets. Control dataset contains all the rest of the data which includes sequences with low complexity, motifs and stable secondary structure.

To further improve our model, we eliminated some data points from the database. Among eliminated data points were RNA sequences that were described to cause artifacts, such as low complexity sequences (GGGG, UUUU, AAAA) and sequences containing motifs (UGGC, GCCA). A strong correlation between the presence of motifs “TGGC” and “GCCA” in siRNAs and reduced cell viability has been found recently (Fedorov et al., 2006). We found that removal of shRNA sequences containing motifs UGGC, GCCA, GGGG, UUUU, and AAAA improved correlation between model predicted and experimental silencing efficiency. Removal of sequences containing other potentially harmful motifs, such as CCCC, did not improve prediction efficiency. It is not always clear why certain motifs are preferred or avoided in efficient siRNA and shRNA molecules. It is assumed that UUUU motif should be avoided during design of shRNAs because it represents an RNA III polymerase termination site. This filtering and the removal of shRNA sequences with low complexity (Morgulis et al., 2006) improved correlation coefficients between model-predicted and experimental silencing score in training and cross-validation datasets. We repeated model training and refitting procedures with the filtered datasets (Figure A2A). During cross validation tests of the improved model, we achieved a Pearson correlation coefficient of 0.51. This value is only slightly smaller than the coefficient that was achieved during model training, which is 0.53. Further improvement of these correlation coefficients was achieved after removal of the shRNA sequences with guide strands capable to form stable self-structures (ΔG < −2 kcal/mol). During cross validation tests of the improved model, we achieved a Pearson correlation coefficient of 0.56. This value is only slightly smaller than the coefficient that was achieved during the model training, which is 0.57 (Figure A2A). This indicates that a very minor model over-fitting occurred. The ROC curves, regression weights, and significance values for variables in the optimal models for complete and filtered databases are shown in Tables 2 and A3 and Figures 2 and A2B.

We are not aware of any other scoring predictive models for miRNA-like shRNAs. A number of models for siRNA predictions that were developed earlier have been tested on miR30 database by Mysara et al. (2011). Table 3 summarizes correlation coefficients that were obtained during these tests. Notable, our model provides higher correlation coefficient (0.56), than other models (maximal correlation coefficient, 0.36), which can be explained by two reasons. First, our model was trained on miR30-based constructs, which are being processed by Dicer and Drosha, unlike chemically synthesized siRNAs which do not require this enzymatic processing for RISC loading. Likely, requirements for enzymatic processing impose additional nucleotide biases at certain sequence positions in efficient silencing shRNA molecules. For example, we found positive correlation between the silencing efficiency of miR30-based constructs and the presence A or U at the 3′ ends of their guide strands. This correlation is negative for chemically synthesized siRNAs and for shRNAs constructs which do not belong to the miRNA-based constructs. Second, we found that elimination of data points that are known to cause experimental artifacts (low complexity and motif sequences) and sequences with stable self-structures from the training set improves overall model performance. We recommend this data filtering step for improved design of efficient silencing miRNA-based molecules.

**Table 3:** **Pearson correlation coefficients for other methods of siRNA silencing predictions**.

**Amarzguioui**	**Hsieh**	**s-Biopredsi**	**i-Score**	**Reynolds**
0.26	0.1	0.34	0.36	0.32
Katoh	DSIR	Thermo21	MysiRNA	siRNA-Scale
0.26	0.35	0.36	0.36	0.36

### Software development

Based on our computational models, we developed the miR_Scan software for prediction of efficient shRNAs for complete mRNA transcripts. miR_Scan allows individual processing of complete list of oligos from input mRNA sequence and produces a list of their scores as an output. When designing the miR_Scan, we took into account the following biological considerations. The miRNA translational suppression is guided by imperfect base pairing between the target and the miRNA guide strand (Doench and Sharp, 2004), and miRNA-like off-targeting effects can result in mRNA translation inhibition and undesirable nonspecific gene down-regulation (Alemán et al., 2007). Specificity of base-pairing depends on the 6–7 base “seed region” at the 5′ end of the guide strand of miRNA. Similarly, most of the unintended mRNA targets share sequence complementarity in their 3′UTR regions with residues 1–8 of siRNA guide strand or contain seed motif matching nucleotides in this region (Jackson et al., 2003; Birmingham et al., 2006; Jackson et al., 2006). The miR_Scan software has a user defined option that allows filtering out shRNAs that contain seed regions of known miRNAs. The list of such regions was extracted from the database of sequences of mature miRNAs http://www.mirbase.org/ftp.shtml. Software also has an option that allows filtering out shRNA candidates with low complexity and undesirable sequence motifs, as discussed above. miR_Scan is available at www.ncbi.nlm.nih.gov/staff/ogurtsov/projects/mi30/ (standalone version of the program is available by request).

Non-overlapping cross validation tests demonstrated a tenfold increase in the frequency of efficient shRNA molecules among the sequences outputted by miR_Scan. Frequency of shRNA molecules with 100% silencing efficiency increased from less than one percent prior to selection to ten percent after selection. miR_Scan high prediction efficiency and hit-rate are due to the use of miR30-based parameters and biology-guided optimization procedure. The implementation of new computational models with shRNA-based features allows us to develop new software for miR30-based shRNA targeting on transcriptome level. According to correlation analysis this approach is more optimal for identification of efficient miR30-based shRNA molecules than methods that were developed for siRNAs.

## Conclusion

We evaluated and optimized parameters for prediction of the silencing efficiency of siRNAs (Shabalina et al., 2006) and shRNAs (this study) and identified universal features for efficient molecules of both types. Comparison of position-dependent consensuses for siRNAs and shRNAs showed that the 5′ ends of both types of small RNAs (1–7 nucleotides) are A/U-rich and form relatively unstable duplexes. Notably, most of these positions are U-rich in siRNAs (Shabalina et al., 2006) and A-rich in shRNAs (Figure 1A). Although the 3′ end of the duplex is G/C-rich in both molecules, the different lengths of these types of molecules do not allow proper alignment of the two consensuses. Similar thermodynamic parameters possess predictive power for design of efficient siRNAs and shRNAs, such as free energy of fully paired duplex and thermodynamic profile (local ΔG) at different duplex positions, specifically at the 5′ end of the duplex. For both types of molecules, stability of predicted secondary structures of small RNA and target mRNA reliably correlated with the silencing efficiency. However, siRNA-specific and shRNA-specific parameters improved silencing predictions in both cases.

Experimental database of miR30-based constructs was used for the optimization of our models and has been incorporated into software. Since all regression weights for shRNA sequence features were particularly tuned to this type of the constructs, it is expected that “miR_Scan” best predictive performance will be achieved with miR30-based constructs. However, taking into account that many optimized parameters in this study correspond to those previously used for siRNA design, it would be promising to test these models for the different sets of miRNA-based constructs.

Our models demonstrated that optimal duplex stability of the fully paired shRNA antisense strand is crucial for the silencing efficiency (Tables 2 and A3). It is clear that for efficient shRNA silencing this parameter should not be too high or too low. Notably, within the optimal shRNA duplex stability range (−35 to −25 kcal/mol), almost all mRNA target sequences are suitable for shRNA–mRNA interactions (Figure A1); this explains why free energy of mRNA target could not influence dramatically the power of the model predictions. This observation is in agreement with the results of previous siRNA design studies (Shabalina et al., 2006). Slow formation of short-living, unstable duplexes does not allow efficient RNA cleavage. On the other hand, GC-rich shRNA antisense strands and target mRNAs are liable to stable self-interactions and formation of stable local secondary structures that compete with shRNA/mRNA target duplex formation. These relationships explain why shRNAs with high duplex stability have low average silencing efficiency (Matveeva et al., 2010) and highlight importance of optimal shRNA-mRNA duplex stability for efficient silencing.

### Conflict of interest statement

The authors declare that the research was conducted in the absence of any commercial or financial relationships that could be construed as a potential conflict of interest.

## Appendix

**Table A1:** **Pearson coefficients of correlation between silencing efficiency and positional nucleotide content**.

**Position**	**A**	**G**	**C**	**U**	**ΔG**
1	0.068	−0.174	−0.162	0.258	0.31
2	0.085	−0.082	−0.08	0.073	0.15
3	0.061	−0.043	−0.048	0.027	0.1
4	0.085	−0.015	−0.058	n.s.	0.09
5	0.078	−0.03	−0.055	n.s.	0.09
6	n.s.	−0.061	n.s.	0.07	0.1
7	0.043	−0.034	−0.055	0.043	0.06
8	n.s.	n.s.	−0.019	n.s.	0.02
9	0.027	−0.046	0.02	n.s.	0.08
10	0.083	−0.062	−0.045	0.021	0.1
11	0.057	n.s.	−0.04	n.s.	0.07
12	0.037	−0.037	−0.039	0.037	0.14
13	0.091	−0.069	−0.1	0.073	0.17
14	n.s.	−0.083	−0.037	0.109	0.07
15	n.s.	n.s.	n.s.	n.s.	n.s.
16	n.s.	−0.03	n.s.	n.s.	0.04
17	n.s.	−0.066	0.013	0.06	0.04
18	n.s.	−0.037	0.021	0.02	n.s.
19	−0.047	−0.019	0.026	0.041	−0.06
20	−0.132	0.047	0.059	0.029	−0.08
21	−0.028	0.032	n.s.	n.s.	0.04
22	0.034	−0.044	−0.031	0.039	−

Correlation coefficients, greater than 0.05, are shown in red. Correlation coefficients, less than −0.05, are shown in blue. n.s., non-significant correlation.

**Table A2:** **Average silencing efficiency according to categorization of the dinucleotide free energy in separate positions of duplex thermodynamic profile**.

**Position of dinucleotide from 5′ end**	**Local duplex stability (kcal/mol)**			
	**−1 ≤ ΔG**	**2 ≤ ΔG< −1**	**−3 ≤ Δ**G**< −2**	**ΔG< −3**
	**AVERAGED SILENCING EFFICIENCY PER BIN**			
1	3.58	1.76	0.55	0.07
2	2	3.03	1.52	0.51
3	1.85	2.17	1.63	0.9
4	2.22	2.09	1.46	1.09
5	1.93	2.43	1.6	0.78
6	1.85	1.99	1.85	0.38
7	1.85	1.48	1.81	0.79
8	1.46	1.65	1.64	1.48
9	1.98	1.64	1.76	0.71
10	1.96	2.18	1.69	0.65
11	2.06	1.67	1.72	0.74
12	2.63	2.44	1.56	0.29
13	2.71	3.18	1.38	0.31
14	1.4	2.41	1.78	0.74
15	1.24	1.44	1.76	1.46
16	1.5	1.69	1.78	1.04
17	1.66	1.86	1.65	1.17
18	1.35	1.71	1.71	1.37
19	0.92	0.95	1.98	1.38
20	0.9	1.02	1.78	1.92
21	1.51	2.13	1.24	1.48

Dark red color highlights average silencing values above 3 (optimal stability). Red color highlights average silencing values above 2 (suboptimal stability). Pink color highlights average silencing values above 1.7 (close to the median stability). Blue color highlights average silencing values below 1 (less than median).

**Table A3:** **Statistics and input parameters for the multiple regression model with efficient prediction of silencing activities on the filtered database (*n* = 10735 oligonucleotides)**.

**Parameter**	**Coefficient**	***P*-value**
Intercept	−69.95	2.442 × 10^−120^
ΔG	−4.712	1.3659 × 10^−91^
ΔG^2^	−0.1204	9.0266 × 10^−90^
ΔG^3^	−0.001024	4.7275 × 10^−91^
ΔG_1_	0.4039	0.00539301
A_1_	1.034	1.3622 × 10^−05^
A_2_	0.8842	9.3786 × 10^−08^
A_3_	0.8345	0
A_4_	0.4935	4.6521 × 10^−12^
A_5_	0.6545	0
A_11_	0.3861	7.211 × 10^−08^
A_l2_	0.5024	4.3667 × 10^−11^
A_13_	1.004	0
A_19_	−0.5374	3.2157 × 10^−14^
A_20_	−1.236	4.1586 × 10^−72^
A_22_	0.2766	0.00019726
G_6_	−0.3111	3.333 × 10^−05^
G_7_	−0.2761	0.0003269
G_9_	−0.3142	1.1835 × 10^−05^
G_10_	−0.851	5.2291 × 10^−23^
G_14_	−0.4395	4.8444 × 10^−09^
G_17_	−0.2894	9.3124 × 10^−05^
G_18_	−0.3296	3.4827 × 10^−06^
G_19_	−0.1936	0.00914666
G_21_	0.3931	3.7168 × 10^−08^
C_4_	−0.2648	0.00034025
C_5_	−0.3841	2.1001 × 10^−07^
C_7_	−0.5525	2.7151 × 10^−13^
C_8_	−0.2283	0.00124906
C_10_	−0.8191	5.6655 × 10^−22^
C_11_	−0.2768	0.00017918
C_13_	−0.3399	7.0633 × 10^−05^
U_1_	2.455	0
U_2_	0.8241	3.3472 × 10^−06^
U_3_	0.7619	0
U_6_	0.6131	0
U_10_	−0.402	6.0954 × 10^−07^
U_12_	0.5706	7.5273 × 10^−14^
U_13_	0.7699	0
U_14_	0.7817	0
U_17_	0.3807	8.677 × 10^−08^
U_22_	0.303	3.6557 × 10^−05^
